# Assessing the risk of obstructive sleep apnoea–hypopnoea syndrome in elderly home care patients with chronic multimorbidity: a cross-sectional screening study

**DOI:** 10.1186/s40064-016-1672-0

**Published:** 2016-01-13

**Authors:** Christos F. Kleisiaris, Evangelos I. Kritsotakis, Zoe Daniil, George Markakis, Ioanna V. Papathanasiou, Zacharenia Androulaki, Konstantinos I. Gourgoulianis

**Affiliations:** Department of Nursing, Technological Educational Institute of Crete, Heraklion, Greece; School of Health and Related Research, University of Sheffield, Regent Court, 30 Regent Street, Sheffield, S1 4DA UK; Department of Respiratory Medicine, University of Thessaly, Larissa, Greece; Department of Nursing, Technological Educational Institute of Thessaly, Larissa, Greece

**Keywords:** Obstructive sleep apnoea syndrome, Chronic diseases, Comorbidity, Multimorbidity, Screening, Epidemiology, General practice

## Abstract

Obstructive sleep apnoea–hypopnea syndrome (OSAHS) and multimorbidity are common in elderly patients, but a potential link between the two conditions remains unclear. This study aimed to assess the prevalence of OSAHS, chronic multimorbidity and their relation in older adults in primary care settings. A screening study was performed in a cross-section of 490 elderly adults (mean age 77.5 years, 51 % male) receiving home care services in Thessaly, central Greece. The Berlin Questionnaire was employed to assess the likelihood for OSAHS and the Epworth Sleepiness Scale to assess daytime sleepiness. Multimorbidity was defined as a documented history of at least two chronic diseases. The prevalence of high risk for OSAHS, excessive daytime sleepiness and multimorbidity was 33.5, 11.6 and 63.9 %, respectively. None of the study subjects had a confirmed diagnosis for OSAHS prior to this study. A marked dose–response association between a high pre-test likelihood for OSAHS and multimorbidity was noted in patients with two [adjusted odds ratio (OR) 3.13; 95 % confidence interval (CI) 1.85–5.30) and three or more (adjusted OR 4.22; 95 % CI 2.55–6.96) chronic morbidities, independently of age, sex and smoking status. This association persisted across different levels for OSAHS risk in the Berlin questionnaire, was insensitive to varying definitions of multimorbidity and more pronounced in patients with excessive daytime sleepiness. These findings point out that primary care physicians who care for elderly patients who present with several, common and burdensome, chronic diseases should expect to find this multimorbidity often coinciding with undetected, and therefore untreated, OSAHS. Thus it is crucial to consider OSAHS as an important co-morbidity in older adults and systematically screen for OSAHS in primary care practice.

## Background

Obstructive sleep apnoea–hypopnoea syndrome (OSAHS) is a common disorder that affects least 2–4 % of the adult population (Young et al. 1993; Epstein et al. 2009). The syndrome affects largely the older adults and its prevalence has been reported to exceed 30 % in those aged 65 years and over (Epstein et al. 2009). Clinically, OSAHS is defined by the occurrence of witnessed breathing (at least five events of apnoea/hypopnoea per hour of sleep) with associated symptoms such as persistent loud snoring and fatigue or excessive daytime sleepiness (Young et al. 2002; Epstein et al. 2009). OSAHS poses an important public health problem not only due to its high prevalence, but also because it is associated with conditions that account for leading causes of morbidity and mortality in adults, including hypertension, metabolic syndrome, cardiovascular and cerebrovascular diseases (Young et al. 2002; Parish et al. 2007; Dorasamy 2007).

OSAHS and chronic diseases are both commonly seen in primary care settings and their frequency varies widely with age and gender (Karachaliou et al. 2007; Minas et al. 2010; Kleisiaris et al. 2014). Multimorbidity—the co-occurrence of several chronic diseases in one individual—is also a common problem encountered in elderly patients (Fortin et al. 2012; Minas et al. 2010; Søndergaard et al. 2015). However, a potential link between OSAHS and multimorbidity has not been studied extensively (Robichaud-Hallé et al. 2012), mainly because the syndrome often remains undiagnosed in primary care practice (Young et al. 1993; Karachaliou et al. 2007). Evidence of an association between OSAHS and multimorbidity could serve as a crucial incentive for the systematic screening for OSAHS in primary care settings (Robichaud-Hallé et al. 2012).

Consequently in this study we sought to assess the risk of OSAHS, measure multimorbidity, and evaluate a possible epidemiological link between the two conditions in an elderly population in primary care settings. We also examined the persistence of this association in the subgroup of subjects who presented with excessive daytime sleepiness and its sensitivity to varying definitions of multimorbidity.

## Methods

### Design and study subjects

In this cross-sectional study we recruited older adults, aged 65 years and older, who were receiving home care services in Thessaly, central Greece. We used a convenience sample of subjects who were attending an ‘Open Care Community Centre for Older People’ or were registered in the ‘Help at Home’ programme (Skaperdas et al. 2010). These health services are directed mainly to rural and semirural populations and include social, nursing and medical care of older adults with a disadvantaged social status and/or a poor family support. We have previously reported the findings of a spirometry program to screen for airflow limitation in the same sample of elderly subjects (Kleisiaris et al. 2014).

Because we had no prior information on the prevalence of OSAHS or multimorbidity in the study population, we used a conservative sample size calculation. We assumed the most conservative scenario of having to estimate prevalence proportions of 50 %. Based on Cochran’s formula, we initially aimed for a minimum sample size of 385 to allow a 5 % level of precision in the estimated prevalence proportions with a confidence level of 95 %. Because we had no prior experience in surveying this particular population, we inflated the sample size to account for a high anticipated non-response rate of 25 % and used a sample size estimate of 518.

Following an initial invitation by the local municipality authorities, 518 elderly adults in sixteen home care settings were approached by a member of the research team, were informed about the study and asked to participate. Screening questionnaires were completed through face-to-face home interviews that lasted between 30 and 60 min. Data were collected during a 6-month period (January–June 2010).

### Screening questionnaires and definitions

We employed the Berlin Questionnaire to screen for OSAHS (Netzer et al. 1999), which has been translated and shown to be a sensitive and predictive screening tool for OSAHS in primary healthcare settings in Greece (Bouloukaki et al. 2013). In brief, the questionnaire evaluates snoring behaviour and witnessed apnoeas during sleep (section 1), tiredness or fatigue after sleep (section 2) and history of hypertension and obesity (section 3). A high pre-test likelihood (“high risk”) for OSAHS is assigned to patients whose responses qualify as high risk in at least two sections of the questionnaire.

As excessive daytime sleepiness (EDS) is a common complaint in OSAHS patients, we used the Epworth Sleepiness Scale (ESS) to assess daytime sleepiness (Johns 1991). ESS quantifies the level of daytime sleepiness by having individuals to self-rate the likelihood of dozing during eight different daytime situations and by producing a summary score that ranges from 0 to 24. A score greater than 10 is indicative of EDS (Johns 1991). We used the validated Greek version of the scale (Tsara et al. 2004).

To record morbidity data, we checked for confirmed diagnoses of chronic diseases in the medical records of each study subject and also assessed current medications. We sought to include common and burdensome chronic diseases based on the main causes of disease burden in primary healthcare in Greece (Minas et al. 2010). We defined multimorbidity as a documented history of at least two chronic diseases (Barnett et al. 2012; Ornstein et al. 2013).

Demographic data were also recorded and included gender, age, weight, height and smoking status. Study subjects with an active smoking status of more than 30 packs per year were considered current smokers and those with no history of active smoking were recorded as non-smokers. Smokers were classified as former smokers if they had quit smoking for at least 12 months. Obesity was defined as a body mass index (BMI) ≥30 kg/m^2^.

### Statistical analysis

Reliabilities of the Berlin questionnaire and the ESS were assessed using Cronbach’s coefficient. 95 % confidence intervals (CI) for proportions were calculated using Wilson’s Score method. The strength and direction of associations between a high pre-test likelihood for OSAHS and the various chronic morbidities were presented using odds ratios (OR) and corresponding CI as calculated by binary logistic regression. The Extended Mantel-Haenzsel Chi square test for linear trend was used to assess the dose–response relationship between the level of multimorbidity and a high pre-test likelihood for OSAHS.

To adjust for potential confounding effects by gender, age and smoking status, multiple logistic regression analysis was performed with a high risk for OSAHS in the Berlin questionnaire constituting the dependent variable and multimorbidity being the main independent variable. Multimorbidity was examined both as a binary (yes/no) and as an ordinal variable (0–1, 2, and 3 or more chronic morbidities).

Because hypertension and obesity are accounted for when assessing the likelihood for OSAHS in the Berlin questionnaire, we performed a sensitivity analysis by excluding these two conditions from the definition of multimorbidity. We also examined the persistence of our findings in the subgroup of subjects with EDS.

All reported *P* values were two-tailed, and a *P* value <0.05 was considered statistically significant. Data were analysed using IMB-SPSS version 22.

### Ethics and consent

This study has a written ethical approval by the Scientific Council of the Department of Medicine at the University of Thessaly (Pr No 5740-7th/October 15, 2008). All study participants were advised about the voluntary nature of the study and gave informed consent.

## Results

### Study subjects

Of the 518 elderly adults solicited, 490 were interviewed and completed fully the study questionnaires (95 % response rate). Study participants had an average age of 77.5 ± 6.9 years (range, 65–98 years), and 51 % were male. None of the study subjects had a confirmed diagnosis for OSAHS prior to this study. Main characteristics of the study participants are presented in Table 1.

**Table 1:** Main characteristics of the 490 study participants

Characteristic	n	Prevalence (%)	95 % CI (%)
Male sex	248	50.6	46.2–55.0
Age (years)			
65–79	314	64.1	59.7–68.2
≥80	176	35.9	31.8–40.3
Smoking status			
Current	44	9.0	6.7–11.9
Former	138	28.2	24.3–32.3
Never	308	62.9	58.5–67.0
Most prevalent chronic morbidities			
Hypertension	257	52.4	48.0–56.9
Obesity	217	44.3	39.9–48.7
Cardiovascular disease^a^	172	35.1	31.0–39.5
Diabetes mellitus	117	23.9	20.3–27.9
Mental health disorder^b^	61	12.4	9.8–15.7
Stroke	24	4.9	3.3–7.2
Depression	21	4.3	2.8–6.5
Number of chronic morbidities			
0–1	177	36.1	32.0–40.5
2	138	28.2	24.3–32.3
3	93	19.0	15.7–22.7
≥4	82	16.7	13.7–20.3
Screening assessment of sleep-related disorders			
Low pre-test risk for OSAHS	238	48.6	44.2–53.0
High pre-test risk for OSAHS	164	33.5	29.4–37.8
EDS	57	11.6	9.1–14.8
EDS and high risk for OSAHS	42	8.6	6.4–11.4

*CI* confidence interval, *OSAHS* obstructive sleep apnoea–hypopnoea syndrome, *EDS* excessive daytime sleepiness

^a^Includes congestive heart failure, coronary artery disease and arrhythmias

^b^Includes anxiety, psychotic disorder and dementia

### Prevalence of OSAHS and multimorbidity

Both the Berlin questionnaire and the ESS demonstrated good degrees of internal consistency (Cronbach’s alpha coefficient, 0.69 and 0.77, respectively). One-third of the study subjects (33.5 %, 95 % CI 29.4–37.8 %) were classified as being at a high risk of having OSAHS according to the Berlin questionnaire. Presence of EDS, as assessed by the ESS, was observed for 57 subjects (11.6 %, 95 % CI 9.1–14.8 %).

The most prevalent chronic morbidities overall were hypertension, obesity, cardiovascular disease, diabetes mellitus and mental health disorder (Table 1). More than two-thirds of all study subjects had one or more chronic conditions, for an average number of 2.1 ± 1.4 morbidities. Defined as the presence of ≥2 chronic diseases, almost two-thirds of subjects were classified as having multimorbidity (63.9 %, 95 % CI 59.5–68.0 %). More than a third of patients had ≥3 chronic morbidities (35.7 %, 95 % CI 31.6–40.1 %). Findings were similar across the different home care services and study locations.

### Association between OSAHS and multimorbidity

A high pre-test likelihood for OSAHS was noted more frequently for study subjects who had a history of stroke, cardiovascular disease, diabetes mellitus or mental health disorder, compared to those with no history of chronic disease (Table 2). Defined as the presence of ≥2 chronic diseases, multimorbidity showed a statistically significant association with a high pre-test likelihood for OSAH (OR 3.67, 95 % CI 2.33–5.76).

**Table 2:** Association between a high pre-test likelihood for OSAHS and main chronic morbidities and multimorbidity

Chronic morbidity^a^	Value	Patients at high risk for OSAHS		Unadjusted effect^b^		Adjusted effect^c^	
		N	Prevalence, %	OR (95 % CI)	*P* value	Adjusted OR (95 % CI)	*P* value
Hypertension	No	45	19.3	1			
	Yes	119	46.3	3.60 (2.40–5.41)	<0.001	3.77 (2.48–5.73)	<0.001
Obesity	No	62	22.7	1			
	Yes	102	47.0	3.02 (2.05–4.45)	<0.001	3.08 (2.06–4.62)	<0.001
Cardiovascular disease	No	99	31.1	1			
	Yes	65	37.8	1.34 (0.91–1.98)	0.136	1.30 (0.87–1.94)	0.198
Diabetes mellitus	No	119	31.9	1			
	Yes	45	38.5	1.33 (0.87–2.05)	0.190	1.29 (0.84–2.00)	0.249
Mental health disorder	No	140	32.6	1			
	Yes	24	39.3	1.34 (0.77–2.33)	0.299	1.40 (0.80–2.45)	0.243
Stroke	No	152	32.6	1			
	Yes	12	50.0	2.07 (0.91–4.71)	0.078	1.88 (0.82–4.31)	0.133
Depression	No	158	33.7	1			
	Yes	6	28.6	0.79 (0.30–2.07)	0.627	0.84 (0.32–2.22)	0.723
Number of chronic morbidities	0–1	30	16.9	1			
	2	53	38.4	3.06 (1.81–5.15)		3.13 (1.85–5.30)	
	≥3	81	46.3	4.22 (2.58–6.91)	<0.001	4.22 (2.55–6.96)	<0.001

*OSAHS* obstructive sleep apnoea–hypopnoea syndrome, *OR* odds ratio, *CI* confidence interval

^a^Table reports most prevalent morbidities

^b^Binary logistic regression

^c^Multiple logistic regression model adjusting for age, sex and smoking status

**Fig. 1:**
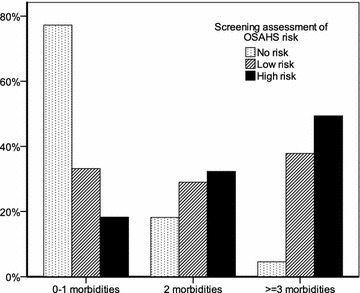
The association between different levels of risk for obstructive sleep apnoea–hypopnoea syndrome (OSAHS) according to the Berlin questionnaire and the level of multimorbidity. Patients whose responses qualified as high risk in at least two sections of the Berlin questionnaire were assigned a high risk for OSAHS, those who qualified in a single section of the questionnaire were assigned to a low risk for OSAHS, and those who did not qualify in any of the sections of the questionnaire were assigned to no risk for OSAHS. The *vertical axis* presents the prevalence of each level of multimorbidity within each level of OSAHS risk

When multimorbidity was treated as an ordinal variable, a statistically significant dose–response association between the level of multimorbidity and a high pre-test likelihood for OSAHS was noted in patients with a history of two (OR 3.06, 95 % CI 1.81–5.15) and three or more (OR 4.22, 95 % CI 2.58–6.91) chronic diseases (χ^2^(1) = 33.34; *P* < 0.001 for linear trend). This association persisted across different levels of risk for OSAHS in the Berlin questionnaire (Fig. 1) and remained unaltered after adjusting for potential confounding effects by age, sex and smoking status in a multiple logistic regression model (Table 2).

### Subgroup and sensitivity analyses

The association between multimorbidity and high risk for OSAHS was more pronounced in the subgroup of the 57 subjects who presented with EDS (adjusted OR 4.77, 95 % CI 1.33–17.18, *P* = 0.017) and retained a dose–response feature (adjusted OR 4.41, 95 % CI 1.05–18.49 for two morbidities; and adjusted OR 5.53, 95 % CI 0.91–33.45 for three or more morbidities).

Following exclusion of hypertension and obesity from the definition of multimorbidity, the association between multimorbidity and high pre-test likelihood for OSAHS retained statistical significance, but was weaker (OR 1.54, 95 % CI 1.04–2.29, *P* = 0.033). When multimorbidity was treated as an ordinal variable, the observed dose–response relationship also retained statistical significance (OR 1.46, 95 % CI 0.93–2.30 for two morbidities; OR 1.72, 95 % CI 0.93–3.18 for three or more morbidities; χ^2^(1) = 4.37, *P* = 0.037 for linear trend).

## Discussion

We assessed the risk of OSAHS, measured the prevalence of multimorbidity and explored the association between the two conditions in a cross-section of older adults in primary care settings. Our findings indicate that caring for elderly patients with sleep apnoea and those with multimorbidity are both common in primary care practice. Importantly, our analysis revealed a marked and persistent dose–response association between multimorbidity and a high risk for OSAHS, independently of age, sex and smoking status.

Even though OSAHS is fairly common, it often remains largely undiagnosed in our populations (Young et al. 1993; Gibson 2004; Karachaliou et al. 2007). Likewise in this study, none of the study subjects had a prior diagnosis for OSAHS but the overall prevalence of subjects at high risk for OSAHS was 33.5 %, which is high and similar to the prevalence reported by previous studies in elderly populations (Sforza et al. 2011).

The prevalence of multimorbidity found in this study is also similar to that reported in epidemiologic studies of older adults in primary care settings around the world (Fortin et al. 2007, 2012). Multimorbidity is an emerging concept in the medical literature and research on its epidemiology and clinical importance is still in its infancy (Fortin et al. 2007; Søndergaard et al. 2015). Consequently, only a limited number of studies have explored a potential link between the presence of multiple morbidities and the occurrence of OSAHS (Smith et al. 2002; Foley et al. 2004; Robichaud-Hallé et al. 2012; Jennum et al. 2013).

The 2003 National Sleep Foundation ‘Sleep in America’ survey explored the association between the prevalence of self-reported sleep problems and chronic diseases and found that older adults (aged 55–84 years) with four or more medical conditions were more likely to have sleep problems (adjusted OR 1.62, 95 % CI 1.05–2.49) than those with no medical conditions (Foley et al. 2004). More recently, Robichaud-Hallé et al. (2012) recruited 120 patients of various ages (30–75 years of age) who presented sleep apnoea of various severities as identified by polysomnography and reported that multimorbidity was associated with severe OSAHS (adjusted OR 7.33, 95 % CI 1.67–32.23). However, the composition of the sample in that study prevented the researches from assessing an exposure–response relationship between OSAHS and multimorbidity (Robichaud-Hallé et al. 2012).

In this study, we found a marked dose–response association between multimorbidity and high risk for OSAHS, which was noted both in patients with two (adjusted OR 3.13, 95 % CI 1.85–5.30) and patients with three or more (adjusted OR 4.22, 95 % CI 2.55–6.96) chronic morbidities and was more pronounced in patients with EDS (adjusted OR 4.77, 95 % CI 1.33–17.18). This association persisted across different levels of OSAHS risk and was insensitive to varying definitions of multimorbidity. Therefore this study adds evidence to a pool of results which suggest that OSAHS is common in older adults and often coincides with their multimorbidity.

Compared to the general population, patients with OSAHS have been shown to use 23–50 % more resources (in terms of physician visits, physician fees and hospitalisations) in the five years prior to their diagnosis (Smith et al. 2002). In a recent controlled study, Jennum et al. (2013) used the National Patient Registry in Denmark to identify all patients diagnosed with sleep-disordered breathing in the country between 1986 and 2006 (over 19,000 patients). They then showed that patients with OSAHS contacted the health care system for various health-related issues and had presented with a wide range of morbidities at least three years prior to their diagnosis of OSAHS. These included such well-known risk associations as obesity, diabetes and cardiovascular disease, but also lesser-known risk associations (Jennum et al. 2013).

As expected we found strong associations between a high pre-test likelihood for OSAHS and the presence of hypertension and obesity in our study subjects. High risk for OSAHS was also more prevalent in patients with stroke, cardiovascular disease and diabetes mellitus in our sample, but these differences were not statistically significant probably because our study was underpowered to detect relatively small effect sizes. There are accumulating evidences suggesting that OSAHS may be an independent risk factor for metabolic syndrome and cardiovascular disorders (Vijayan 2012). However, many well-known risk factors for OSAHS, such as age, male sex, obesity, diabetes and hypertension are also known to increase the risk for cardiovascular disease and/or metabolic syndrome (Vijayan 2012). Similarly, a relation between OSAHS and stroke seems almost inevitable given the association of OSAHS with hypertension. Establishing such cause-effect relationships with OSAHS is difficult, requires more data from intervention studies and randomized controlled trials and remains an active area of research.

It has been suggested that sleep-related symptoms such as snoring and excessive daytime sleepiness are associated with anxiety and depression (Bouscoulet et al. 2008). In our screening assessment, we found that patients with a history of depression or other mental health disorder were no more likely to present with a high-risk of OSAHS or EDS than were patients without depression. This is in agreement with a recent cross-sectional study on adults diagnosed with OSAHS at the sleep laboratory which did not find any evidence to indicate an independent association between the severity of sleep apnoea and the incidence of depression (Rezaeitalab et al. 2014).

This study has limitations that should be acknowledged. First, the cross-sectional nature of our study does not allow conclusions to be drawn regarding cause-effect relationships. Second, measurement error is possible in our assessment of multimorbidity because chronic morbidities were documented from medical files and we cannot exclude the possibility of undocumented or undiagnosed medical conditions. Third, performing a full sleep investigation including overnight in-laboratory polysomnography was not feasible in this large population-based study, as it is too costly, not possible for all study subjects, and portable equipment is unreliable. Instead we used two highly sensitive screening questionnaires to assess sleep apnoea, but these may be less specific for the diagnosis of OSAHS in older adults (Sforza et al. 2011). Therefore we cannot exclude the possibility that some subjects without sleep-disordered breathing may have been falsely classified as having a high risk for OSAHS in this study. Moreover, we don’t know whether central sleep apnoea or other types of sleep-disordered breathing might be present in our sample. It is difficult to assess the effect that such differential misclassifications might have had in our results; they might have given rise to falsely high or falsely low odds ratios in this study. Nevertheless, robustness of our findings is supported by our sensitivity and subgroup analyses.

The present study adds to an emerging pool of information regarding two newly developing areas of research, i.e. the epidemiology and clinical importance of OSAHS and those of multimorbidity. Comorbidity should be regarded as a patient characteristic that may affect the occurrence and relevant outcomes of another disease. Instead of focusing mainly on specific chronic diseases and their specific outcomes, this study points out that future research may need to consider OSAHS as an important co-morbidity in older adults.

This burden needs to be addressed with policies aiming at improving the identification of undiagnosed OSAHS via population screening in primary care (Young et al. 2002). As snoring and sleepiness, the hallmarks of OSAHS, are highly prevalent in the general population, the challenge for primary care providers will lie in determining which patients with these symptoms warrant further evaluations (Young et al. 2002). The present study points out that practitioners caring for elderly patients who present with several, common and burdensome, chronic diseases should expect to find this multimorbidity often coinciding with undetected, and therefore untreated, OSAHS. If OSAHS is confirmed, it may affect the management of the patient who could benefit from treatments that are known to help control many associated chronic conditions (Robichaud-Hallé et al. 2012).

Multimorbidity may also be an important methodological issue in studies which assess the effects of new treatments or other interventions on clinical outcomes of elderly patients with OSAHS. Randomised controlled trials that exclude patients with multimorbidity may have severely limited external validity. Observational studies should adjust for potential confounding effects of multimorbidity on outcome variables in patients with OSAHS.

## Conclusions

OSAHS is prevalent and largely undiagnosed in older adults and often coincides with multiple, common and burdensome, chronic diseases. This is a crucial incentive to consider OSAHS as an important co-morbidity in older adults and systematically screen for OSAHS in primary care practice.
